# Composite Diffuse Large B-cell and Mantle Cell Lymphoma: A Case Report

**DOI:** 10.7759/cureus.963

**Published:** 2017-01-08

**Authors:** Farhan Mohammad, Gwenalyn Garcia, Shiksha Kedia, Juan Ding, Matthew Hurford, Alexander Bershadskiy

**Affiliations:** 1 Department of Hematology and Oncology, Staten Island University Hospital; 2 Department of Hematopathology, Staten Island University Hospital

**Keywords:** composite, lymphoma, r-chop

## Abstract

Composite lymphoma is an extremely rare clinical entity and is characterized by the presence of two different subtypes of lymphoma in the same lymph node. We report a case of composite lymphoma in a 57-year-old male presenting with leg and groin pain. The right inguinal lymph node biopsy showed large and small cells. Immunohistochemistry was consistent with large cells staining for diffuse large B-cell lymphoma (DLBCL) and small cells positive for mantle cell lymphoma (MCL). Polymerase chain reaction (PCR) analysis of immunoglobulin heavy (IGH) chain and immunoglobulin kappa (IGK) light chain gene rearrangements confirmed that the two were clonally unrelated neoplasms. There are only two reported cases of composite lymphoma with this combination in the published English literature. We report the third such case and discuss the pathology, diagnostic challenges and management of composite lymphoma.

## Introduction

Composite lymphoma is defined by the presence of two distinct architectural and cytological subtypes of lymphoma occurring within the same lymph node. The majority of reported composite lymphomas represent two forms of non-Hodgkin lymphoma (NHL), e.g., mixed small and large cell lymphoma. The rare association we discuss is the presence of Hodgkin lymphoma and NHL in the same lymph node. It is believed that Hodgkin lymphoma and NHL represent mutually exclusive clinicopathological entities. Herein, we present a case of composite lymphoma consisting of diffuse large B-cells and small cells of mantle cell lymphoma.

Consent was obtained from the patient for the publication of this report.

## Case presentation

A 57-year-old male with a history of hypertension and benign prostatic hypertrophy presented with complaints of decreased appetite, leg cramps with associated discomfort in his right groin and swelling in the right leg. The patient also reported fatigue with a 10-pound weight loss over the preceding month. He denied fever, chills or night sweats. On physical examination, the spleen was palpable one finger breadth below the costal margin with mild adenopathy in the right and left inguinal regions. Pitting edema in the left lower extremity was present. A computed tomography (CT) scan of the abdomen and pelvis revealed a large conglomeration of lymph nodes involving the right periaortic region extending to the right pelvic side wall with areas of central necrosis. Also seen was a right inguinal lymph node measuring about 2.8 x 2.1 cm and splenomegaly. A CT scan of the chest showed extensive mediastinal adenopathy measuring up to 1.3 x 1.4 cm and bilateral axillary lymphadenopathy with the largest lymph node measuring 1.5 x 1.1 cm.

Initial blood work showed a lactate dehydrogenase (LDH) level of 634 U/L, white cell count of 7.36 million cells/mcL with a normal differential, hemoglobin of 11.9 g/dL and a platelet count of 276,000/microliter. Coagulation studies and a comprehensive metabolic panel were within normal limits.

**Figure 1:**
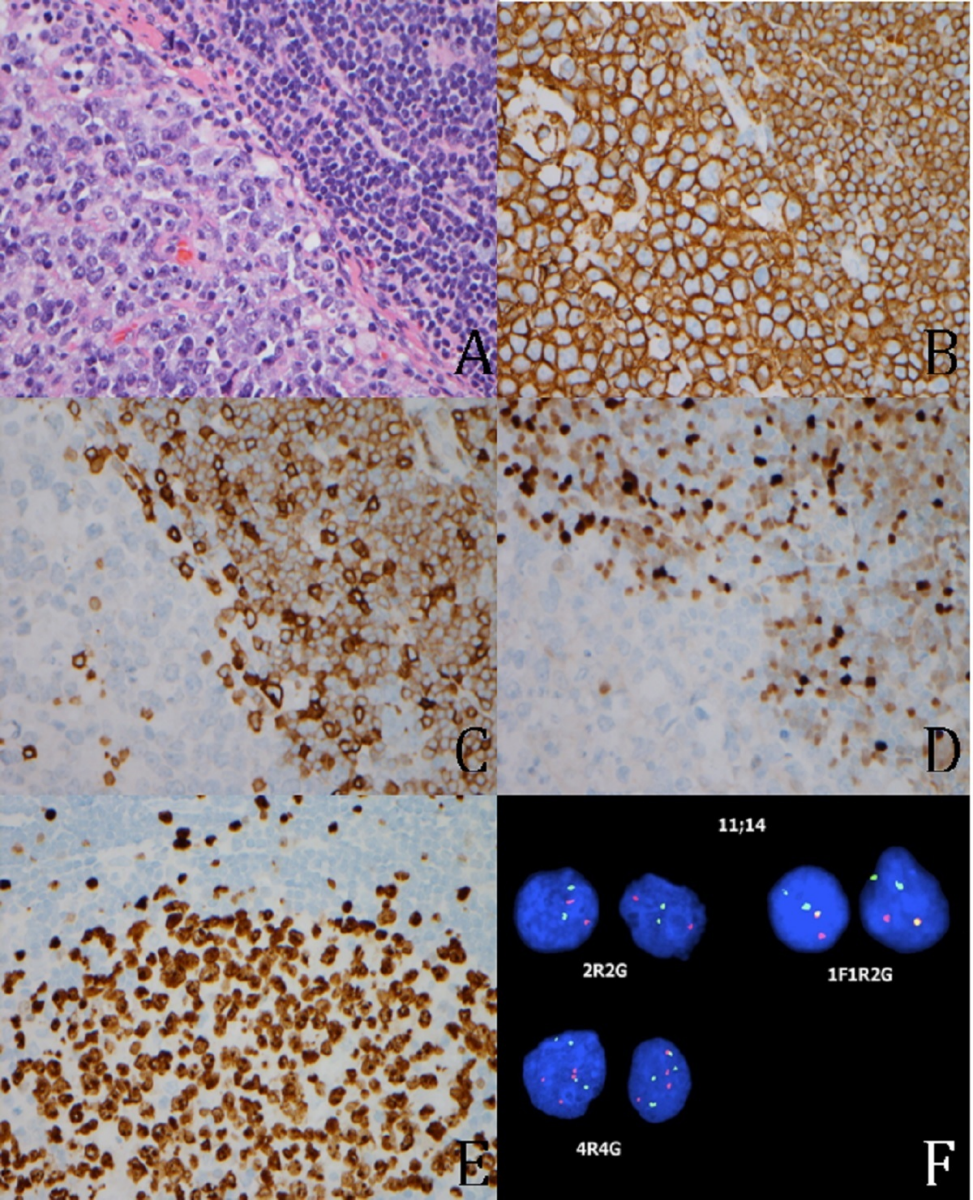
Lymph Node biopsy A. Lymph node biopsy (400x) showing the transition between the small lymphocytes of MCL (upper right) and the large lymphocytes of DLBCL (lower left). B. The small cells of MCL (upper right) are positive for CD20 (400x). The large cells of DLBCL (lower right) are positive for CD20 (400x). C. The small cells of MCL are positive for CD5 (400x). D. The small cells of MCL are positive for cyclin D1 (400x). E. Low Ki-67 proliferation rate of MCL is seen in the upper part of the picture (440x). High Ki-67 proliferation rate of DLBCL is seen in the lower part of the picture (440x). F. The yellow fusion signal of IGH-CCND1 translocation by FISH is present in the MCL (1F1R2G), as are additional gains of cyclin D1 (4R4G).

An excisional biopsy of the right inguinal lymph node was performed, and pathologic exam revealed a biphasic lymphoid population consisting of a large cell population strongly positive for B-cell antigens, CD20 and PAX5, and germinal center cell antigens, CD10 and BCL6. Ki-67 was expressed in 80-90% of the cells. The cell regulatory protein p53 was expressed in the majority of the larger cells, supporting the diagnosis of germinal center-like large B-cell lymphoma. Unexpectedly, the small cell component was also positive for the B-cell antigens, CD20 and PAX 5, with aberrant co-expression of the T-cell antigen CD43 (dim). Distinct nuclear positivity for cyclin D1 (BCL1) was present. BCL2 was also positive, consistent with a diagnosis of mantle cell lymphoma. Epstein-Barr encoding region (EBER) in situ hybridization was negative (Figure 1).

To confirm the presence of two clonally unrelated neoplasms rather than large cell transformation of MCL, PCR analysis of IGH chain and IGK light chain gene rearrangements were performed on the two components after macrodissection. Fluorescent in situ hybridization (FISH) analysis was positive for rearrangement of CCND1/IGH in 13% of the cells and multiple gains of chromosomes resulting from the t(11;14) (q13;q32) translocation, which is most commonly associated with MCL. Hence, we reached the final diagnosis of B-cell lymphoma CD10 positive (80-90% large cell lymphoma) and MCL (10-20%).

Subsequently, the patient underwent a positron emission tomography-computed tomography (PET-CT) scan which revealed extensive fluorodeoxyglucose-avid (FDG-avid) lymphadenopathy above and below the diaphragm with a maximum standard uptake value (SUV) of 39.8 measured at the 15 x 7.5 cm conglomerate of lymph nodes within the right pelvic sidewall. Multiple FDG-avid lesions were also seen within the liver and spleen along with a right rib lesion.

Bone marrow biopsy and aspirate findings were consistent with mild hypercellularity (50-60%) with increased interstitial lymphocytes, predominantly small to medium in size with irregular nuclei and distinct nuclear positivity for cyclin D1 (BCL1). Immunohistochemistry and FISH studies were consistent with mantle cell lymphoma with no evidence of large cell lymphoma. Further studies included cerebrospinal fluid (CSF) analysis, which was negative for malignant cells. Multigated acquisition (MUGA) scan showed an ejection fraction of 68%. The patient was treated with six cycles of a chemotherapy drug combination of rituximab, cyclophosphamide, hydroxydaunomycin, Oncovin® and prednisone (R-CHOP), along with four doses of intrathecal methotrexate given with the first four cycles of chemotherapy. A central nervous system (CNS) prophylaxis was administered because of the presence of visceral and rib disease. A repeat PET-CT scan after the completion of chemotherapy showed resolution of the previously FDG-avid lesions, and bone marrow biopsy was negative for the presence of any lymphoproliferative disorder. The patient is currently on consolidation chemotherapy and has received two cycles of cytarabine to be followed by high-dose methotrexate. He is planned to undergo an autologous stem cell transplantation following consolidation.

## Discussion

The term composite lymphoma refers to two or more distinct types of lymphoma coexisting in the same lymph node or organ. These entities are rare, occurring in about one to 4.7% of all lymphomas [1]. A variety of composite lymphomas have been described in the medical literature, including combined Hodgkin lymphoma (HL) and non-Hodgkin lymphoma (NHL), combined B-cell and T-cell NHLs, and combined B-cell NHL of different types. In cases of combined B-cell NHL or combined NHL and HL, the distinct lymphoma subtypes comprising the composite lymphoma may either be clonally related or unrelated [2-3].

DLBCL is the most commonly occurring NHL, accounting for about one-third of total cases. MCL, on the other hand, is less common and accounts for about four percent of cases [4]. Both have been described separately as individual components of composite lymphoma in multiple case reports and series. However, to our knowledge, only two cases of composite lymphoma involving DLBCL and MCL together have previously been described.

Ho et al. reported two elderly male patients diagnosed with advanced-stage NHL. Lymph node biopsies in each of the cases showed a dominant population of large cells along with a minor population of small lymphocytes. Both populations of cells were CD20 positive; however, only the small cell population expressed cyclin D1 and tested positive for the t(11;14) CCND1/IGH translocation characteristic of MCL. Similar to the present case, both previously reported cases had bone marrow involvement with MCL but not DLBCL [5-6].

Given the rarity and heterogeneity of composite lymphomas, optimal treatment strategies have not been well defined. In formulating the therapeutic plan, both distinct lymphoma entities must be taken into consideration, albeit targeting the more aggressive lymphoma is prioritized [2]. DLBCL usually presents with an aggressive clinical course; however, cure is achieved in about 60% of patients with advanced disease treated with R-CHOP. MCL, on the other hand, is considered a relatively aggressive form of lymphoma, but it is characterized by the incurability typical of indolent lymphomas. Management options range from observation (for patients with clinically indolent disease with favorable pathologic features) to aggressive regimens such as hyper-CVAD (cyclophosphamide, vincristine, doxorubicin and dexamethasone) plus rituximab. For eligible patients, first line consolidation with high-dose therapy and autologous stem cell transplant is recommended [7-9].

In the series by Ho et al., the first patient reported received six cycles of R-CHOP, initially achieving a complete response and subsequently relapsing with DLBCL 15 months after completion of therapy. The second patient declined combination chemotherapy and was treated with single-agent rituximab, achieving stable disease [5]. In line with current treatment strategies, our patient received rituximab with chemotherapy and is planned for consolidation therapy followed by autologous stem cell transplant [8-9].

## Conclusions

Composite lymphoma is a rare occurrence, and it is imperative that a diagnosis of lymphoma be made with an excisional biopsy of the lymph node, rather than fine-needle aspiration or core biopsies. A meticulous examination of the lymph node should be done to identify different cell types; their clonality should be tested with in situ hybridization technique; and management should be directed toward the higher grade of the histology.
